# Sustainable biorefining and bioprocessing of green seaweed (*Ulva* spp.) for the production of edible (ulvan) and non-edible (polyhydroxyalkanoate) biopolymeric films

**DOI:** 10.1186/s12934-023-02154-7

**Published:** 2023-07-31

**Authors:** N. Arul Manikandan, Piet N. L. Lens

**Affiliations:** 1 National University of Ireland Galway, Galway, H91 TK33 Ireland; 2 DCU Glasnevin Campus, Dublin City University, Dublin 9, Ireland

**Keywords:** Biorefining, Bioprocessing, Dark fermentation, Ulvan, Polyhydroxybutyrate

## Abstract

A sustainable biorefining and bioprocessing strategy was developed to produce edible-ulvan films and non-edible polyhydroxybutyrate films. The preparation of edible-ulvan films by crosslinking and plasticisation of ulvan with citric acid and xylitol was investigated using Fourier transform infrared (FTIR) spectroscopy and differential scanning calorimetry (DSC) analysis. The edible ulvan film was tested for its gut-friendliness using *Lactobacillus* and *Bifidobacterium* spp. (yoghurt) and was shown to improve these gut-friendly microbiome's growth and simultaneously retarding the activity of pathogens like *Escherchia coli* and *Staphylococcus aureus*. Green macroalgal biomass refused after the extraction of ulvan was biologically processed by dark fermentation to produce a maximum of 3.48 (± 0.14) g/L of volatile fatty acids (VFAs). Aerobic processing of these VFAs using *Cupriavidus necator* cells produced 1.59 (± 0.12) g/L of biomass with 18.2 wt% polyhydroxybutyrate. The present study demonstrated the possibility of producing edible and non-edible packaging films using green macroalgal biomass as the sustainable feedstock.

**Graphical Abstract:**

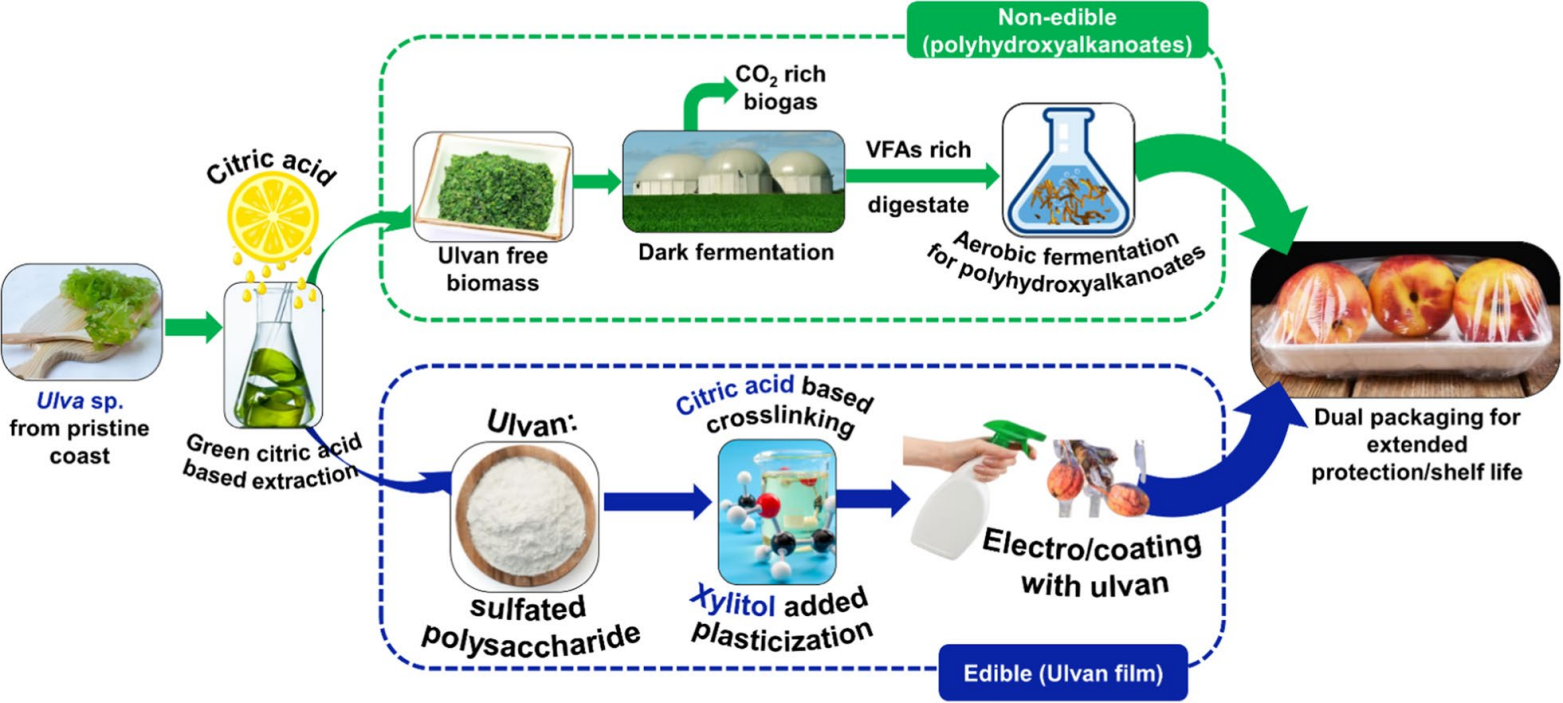

## Highlights

- Novel bioprocess to produce edible and non-edible films from *Ulva* sp. was developed.
- Edible (ulvan) film was formed by citric acid crosslinking and xylitol plasticisation.
- Edible film boosted the growth of gut-friendly microbes and retarded pathogen growth.
- Dark fermentation was applied to produce volatile fatty acids using residual biomass.
- Polyhydroxyalkanoates were produced by *Cupriavidus necator* using volatile fatty acids.

## Introduction

Annual petroleum based plastic production touched 360 million tonnes in 2018 globally, amongst which 22 to 43% of the polymers are disposed of in landfills, and 10–20 million tons of plastics are dumped into the ocean each year [1]. With 42% of global plastic production utilised for packaging, the packaging sector is the largest consumer of petroleum based plastic [2]. It is suggested that the continuous use of single-use plastic and its improper disposal is the key reason for global plastic pollution [2]. However, proper food packaging is inevitable to ensure high food safety and extended product shelf-life [3, 4].

Therefore, contemporary research focuses on developing fully bio-based packaging films with complete biodegradability, enhanced properties, and food safety. Edible packaging films like starch and ulvan are researched and developed that typically use sustainable and biodegradable materials to wrap or coat around the food, which generates no waste [5]. Currently, multiple packaging film products like Tetra Pak^®^ are handled to enhance food protection and extend the shelf life [6]. Thus, an added sturdy packaging film is inevitable in addition to the edible bio-packaging film. Preparing external packaging shells using biopolymers like polylactic acid (PLA) or polyhydroxyalkanoates (PHAs) make the food packaging biodegradable. Sourcing these edible and non-edible packaging films from renewable materials, as e.g. seaweed, makes the packaging biodegradable and sustainable.

The use of seaweed as a feedstock for biopolymers is intriguing as it is one of the most abundant sea resources which does not compete with food crops over land and water [7]. *Ulva* spp. is particularly attractive among all seaweed species as a potential biomass feedstock due to its rapid growth rate and exceptional biomass composition made of ulvan and monosaccharides, viz. hexose and pentose sugars [8]. While ulvan is a possible precursor for edible packaging films, the remaining hexose, pentose and protein fraction from ulvan-free biomass could be aerobically fermented to produce PHAs. Thus, *Ulva* spp. Can be used as a sole feedstock for making edible ulvan films and non-edible polyhydroxyalkanoate films. Ulvan is a sulfated polysaccharide usually soluble in water and composed of rare molecules like rhamnose and uronic acids, which are helpful in food supplements and biomedical applications [9, 10]. Polyhydroxyalkanoates are a sustainable material with outstanding mechanical properties and possess the unique capability to degrade in marine and soil environments [11, 12].

Very few literature reports have been published regarding the preparation of edible ulvan films [13]. For instance, ulvan was isolated by Guidara et al. [10] and plasticised using varying concentrations of sorbitol and glycerol. Later, ulvan smart films were prepared and analysed for their optical, thermal and mechanical properties [14]. Recently, our lab showed that the citric acid-based crosslinking of ulvan could render ulvan insoluble in water [9]. Therefore, the present study improved the citric acid crosslinked ulvan films by plasticising them with xylitol. Citric acid [9, 15] and xylitol [16] have been used as crosslinking and plasticising agents to produce edible cellulosic [15], starch and protein-based [2, 17] composite films.

Likewise, there have been few attempts at hydrolysing the *Ulva* spp. to produce polyhydroxyalkanoates. Gosh et al. [1] used subcritical hydrolysis to convert whole *Ulva* biomass as a substrate for *Haloferax mediterranei* to produce polyhydroxyalkanoates in pneumatically agitated bioreactors. Banu et al. [18] used a chemo thermal coupled sonic homogenisation technique for volatile fatty acid recovery from *Ulva faciata,* and obtained a maximum volatile fatty acids (VFAs) production of 2172 mg/L. All the literature on using *Ulva* spp. for polyhydroxyalkanoates production has used the entire *Ulva* biomass, including ulvan. While the studies focusing on ulvan-based packaging film production discarded the ulvan-free biomass generated during the ulvan extraction process. Thus, there is a demand for utilising both ulvan and ulvan-free biomass as single-handed feedstock for producing, respectively, edible and non-edible packaging films.

Novelty of the present study relies on the biorefining of *Ulva* spp. to fractionate ulvan from ulvan-free biomass by extraction and bioprocessing them to form edible ulvan films and non-edible polyhydroxyalkanoates films. Thus, the present study focuses on the biorefining of ulvan from *Ulva* spp. and edible ulvan films were prepared by crosslinking with citric acid and plasticising with xylitol. The biomass devoid of ulvan (ulvan-free) was digested using a dark fermentation strategy to produce volatile fatty acids. Finally, VFAs produced during dark fermentation, was used as a substrate for *Cupriavidus necator* to produce PHAs.

## Materials and methods

### Source of* Ulva* spp., chemicals and microorganisms

*Ulva* spp. biomass used in this study was collected on 9th August 2021 from the Spiddal coast (53° 24′ 00.6″ N-9° 31′ 20.2″ W), Galway, Ireland. *Ulva* spp. biomass was brought into the lab on the same day and washed abundantly with tap water to remove any foreign matter, soil and salts adhered to this biomass. Thereafter, the *Ulva* spp. was squeezed manually to remove excess water, and further, the *Ulva* spp. was frozen at − 80 °C for two days and then freeze-dried. All the freeze-dried samples were stored at − 40 °C and recovered on the day of the experiment. Citric acid and xylitol used for crosslinking and plasticisation were obtained from Sigma-Aldrich (Steinheim, Germany), absolute ethanol was purchased from Lennox^®^ (Dublin, Ireland), commercial ulvan, polyhydroxybutyrate and polyhydroxybutyrate valerate was purchased from Carbosynth^®^ (Newbury, United Kingdom), Sigma-Aldrich (Steinheim, Germany) and Goodfellow^®^ (Cambridge, United Kingdom). Hydrochloric acid, sulfuric acid, sodium carbonate and sodium hydroxide used in this study were procured from Alfa Aesar® (Kandel, Germany). Finally, nutrient broth and nutrient agar was purchased from Merck (Darmstadt, Germany).

*Cupriavidus necator* (DSMZ 515) used in the study for polyhydroxybutyrate production was procured from DSMZ (Braunschweig, Germany), and the culture was retrieved in nutrient agar. A single colony from the agar plate was transferred into 100 ml nutrient broth in a 250 ml Erlenmeyer flask, which was aerobically cultivated at 28 °C for 24 h in an orbitary shaker (Brunswick Scientific innova 44 incubator orbital shaker, Eppendorf, Germany) agitated 200 rpm. Thereafter, 1 ml of glycerol stocks were prepared with 15% glycerol concentration and stored at − 80 °C until further use. Basal mineral medium from Himedia Ltd. (Einhausen, Germany) was added as the mineral supplement in VFAs-rich medium for *C. necator* cultivation*.* The anaerobic granular sludge (AGS) biomass used in the present study for VFAs production via dark fermentation was obtained from a dairy wastewater treating bioreactor located at Kilconnel (Ireland). The AGS biomass consisted of a total solid content of 37.8 g/kg; the same value was previously reported by Logan et al. [19]. The gut-friendly microbiome used in the present study was taken from live yoghurt cultures obtained from Dunnens Dairy sold at Aldi Stores Ltd. (Galway, Ireland). *Escherchia coli* and *Staphylococcus aureus* were kindly provided by the Enteric Pathogen Research Laboratory (Department of Microbiology, University of Galway, Ireland). Milli-Q water with an average conductivity of 18 MΩ cm^−1^ was used throughout the study.

### Biorefining of *Ulva* spp. biomass for fractionation of ulvan from ulvan-free biomass

Ulvan extraction from *Ulva* spp. was carried out as previously optimised by Manikandan et al. [9]. For this extraction, 30 g/L of *Ulva* spp. biomass was added with 1 wt% of extractant, i.e. citric acid. The extraction was carried out for four hours in a 250 mL screw-capped bottle placed horizontally on a shaking water bath incubated at 90 °C and agitated at 100 rpm. At the end of the experiment, the solution was centrifuged at 6000 × g for 15 min, the supernatant was gently decanted into a new bottle, and the *Ulva* biomass devoid of ulvan was preserved at − 40 °C for dark fermentation experiments (see “Dark fermentation of ulvan-free Ulva spp. biomass for VFAs production” section). Three times the volume of ice-cold ethanol was added to the supernatant, and a white precipitate containing ulvan was obtained. Ulvan precipitate obtained by adding ethanol was centrifuged at 6000 × g for 15 min. The white pellet obtained after centrifugation was dried at 70 °C in a hot-air oven until a constant weight was observed in a weighing balance [9]. The quality of the thus obtained ulvan was compared with commercial ulvan using FTIR and proton-NMR analysis (see “Analytical techniques” section).

### Preparation and characterisation of edible ulvan films

Extracted ulvan (see “Biorefining of Ulva spp. biomass for fractionation of ulvan from ulvan-free biomass” section) was used to prepare edible ulvan films using a simple solution casting method. Three different ulvan films were prepared by dissolving 3 g of ulvan alone in 100 mL milli-Q water [14], 3 g of ulvan plasticised with 0.6 g (20 wt% of ulvan) of xylitol in 100 mL milli-Q water, and 3 g of ulvan plasticised with 0.3 g of xylitol (10 wt% of ulvan) and 0.3 g of citric acid (10wt% of ulvan) in 100 mL milli-Q water. All three solutions were stirred using a magnetic stirrer at 250 rpm for 12 h under ambient (20 °C) conditions. At the end of the experiments, three different solutions were gently transferred into glass Petri dishes without forming any bubbles. All the Petri dishes were dried at 80 °C overnight to create films [15]. The films were peeled off from the plates. The films containing ulvan, those containing ulvan plasticised with xylitol, and those with ulvan plasticised with xylitol and crosslinked with citric acid were named Ulvan, Ulvan-Xyl, and Ulvan-Xyl-CA, respectively.

All films were characterised using FTIR described below (see “Analytical techniques” section). Further, the gut-friendliness and antimicrobial activity of the three different ulvan films were tested by growing a gut-friendly microbiome and pathogens (*S. aureus* and *E. coli*). All microbiological analyses were carried out in 15 mL test tubes containing 9 mL of nutrient broth, 0.1 g of respective films and 1 mL of respective microbial cultures as the inoculum. Before inoculation, all tubes containing medium and films were sterilised using an autoclave at 121 °C for 15 min. Experiments were carried out in a temperature-controlled orbitary shaker agitated at 130 rpm and incubated at 37 °C for 48 h [20, 21]. Microbial culture growth was measured by recording the increase in optical density (600 nm) using a UV–Visible spectrophotometer (UV-1900 Shimadzu, Kyoto, Japan). All analyses were carried out in triplicates, and the results are reported as mean ± standard deviation. Further, the difference between the two data sets and their statistical significance were calculated by employing Tukey's test using Origin 2018 software (v. 9.5.95, Northampton, USA). Changes in the microbial community of the gut-friendly microbiome in the presence of the three different edible films, viz. Ulvan, Ulvan-Xyl and Ulvan-Xyl-CA were analysed using microbial community analysis, as described in “Microbial community analysis” section.

### Dark fermentation of ulvan-free *Ulva* spp. biomass for VFAs production

Ulvan-free biomass discarded after ulvan extraction and consecutive centrifugation (see “Biorefining of Ulva spp. biomass for fractionation of ulvan from ulvan-free biomass” section) was used as a substrate for dark fermentation. The dark fermentation experiments were carried out in 150 mL serum bottles containing 2.5 g dry ulvan-free biomass suspended in 85 mL milli-Q and 15 mL (0.5 g VSS) AGS biomass mentioned in “Source of Ulva spp., chemicals and microorganisms” section. The AGS biomass used in the dark fermentation experiments was heat treated for 30 min on a hot plate after attaining 100 °C [22]. Inoculum pretreatment was done to avoid the presence of methanogens and enrich hydrogenogens in the seed culture [23]. A substrate overloading strategy (inoculum/substrate ratio of 1:5) and longer incubation time (five weeks) were permitted to divert the evolved biohydrogen to VFAs production [24].

The dark fermentation experiments were carried out at a solution pH of 7 in triplicates in an orbitary shaker agitated at 120 rpm and 37 °C [22]. Before inoculation, the bottles containing substrate were purged with nitrogen for 15 min to remove dissolved oxygen and create an anaerobic atmosphere. For five weeks, gas and liquid samples were withdrawn at the end of every week and analysed for their composition using gas and liquid chromatography, respectively (see “Analytical techniques” section). Microbial community analysis was carried out at the end of the dark fermentation experiments (week 5), as described in “Microbial community analysis” section.

### Cultivation of *C. necator* on VFAs for polyhydroxybutyrate production

The liquid fraction of the digestate from the dark fermentation experiments was harvested by centrifuging the contents of the serum bottles at 5000 rpm for 15 min. Thereafter, the liquid fraction of the digestate rich in VFAs was used as the sole carbon source for *C. necator* cultivation. However, the essential minerals needed for *C. necator* were supplemented with the basal mineral media supplied by Himedia^®^ laboratories. The basal mineral media composition was as follows (g/L): ammonium chloride, 0.8; dipotassium phosphate, 0.7; magnesium sulfate heptahydrate, 0.01; disodium EDTA, 0.0092; ferrous sulfate heptahydrate, 0.007; calcium sulfate dihydrate 0.002; boric acid, 0.0001; zinc sulfate heptahydrate, 0.0001; manganese sulfate tetrahydrate, 0.00002; cobalt nitrate, 0.00001, sodium molybdate dihydrate, 0.00001 and copper sulfate pentahydrate, 0.0005.

Since the liquid fraction of the digestate originating from the dark fermentation comprised a variety of VFAs, in addition to using digestate as the carbon source, separate experiments incorporating each VFA, viz acetic, propionic, butyric, iso-butyric, valeric, iso-valeric and caproic acid, were tested for their performance in *C. necator* growth and PHB accumulation. All experiments were conducted aseptically under aerobic conditions in a 150 mL baffled conical flask containing a 50 mL working volume and 10% (v/v) inoculum. pH of the medium was adjusted to 7 (± 0.5) by adding sodium carbonate, and the temperature and agitation were maintained at 28 °C and 200 rpm in an orbitary shaker, respectively [20]. All the experiments were carried out in triplicates and lasted for 4 days.

Samples from all the bottles are withdrawn at an equal interval of 12 h and analysed for optical density (OD_600_) and total carbon concentration using a UV–Visible spectrophotometer (UV-1900 Shimadzu, Kyoto, Japan), and total organic carbon analyser (TOC-L CSN Analyser, Shimadzu, Japan) [20]. The specific growth rate was estimated from the *C. necator* growth kinetics using the following Eq. 1 [20, 25]:

1 $$\mu = \frac{1}{x}\times \frac{dx}{dt}$$

where μ and x are the specific growth rate (h^−1^) and biomass concentrations (g/L), respectively.

Biomass and polyhydroxybutyrate concentrations were measured only at the end of the experiments. Biomass concentration was estimated by harvesting the entire *C. necator* cells from the conical flask using centrifugation spinned at 6000 × g for 10 min. The supernatant in the centrifuge bottles was gently discarded, and the bacterial pellet was dried overnight at 60 °C in a hot air oven to obtain the biomass concentration in g/L [20].

For PHB estimation, the 1 ml aliquots of the sample containing bacterial cells were digested using 98% sulfuric acid for 1 h in a boiling water bath. This process converts polyhydroxybutyric acid to crotonic acid, which is measured with a UV–Visible spectrophotometer at 235 nm using pure sulfuric acid as the reference [20, 26] and a standard curve was constructed using commercial PHB. All the experiments were carried out in triplicates; therefore, the results are presented as Mean ± Standard deviation. The quality of the polyhydroxybutyrate extracted from the *C. necator* biomass was compared with commercially available polyhydroxybutyrate (PHB) and polyhydroxy-valerate (PHBV) using FTIR, proton-NMR and DSC analysis (See “Analytical techniques” section). However, for proton—NMR analysis, the polymer samples were dissolved in deuterated chloroform (CdCl_3_) instead of deuterated water used for dissolving ulvan [20].

### Analytical techniques

#### Film characterisation

FTIR analysis was carried out under attenuated total reflectance (ATR) mode using a FT-IR spectrometer (PerkinElmer Spectrum 400, Waltham, Massachusetts) fitted with a diamond (Diamond/ZnSe) ATR attachment [9]. An average of 50 scans were measured in the range of 400 to 4000 cm^−1^ after placing a few milligrams of respective samples on the crystal plate and gently pressing it with a flat-tip plunger attached to the ATR accessories. For proton—Nuclear Magnetic Resonance (NMR) analysis, 5 ml of respective ulvan samples were dissolved in deuterated water (D_2_O) and transferred into NMR tubes [9]. NMR tubes containing samples were placed in a 600 MHz NMR instrument equipped with a cryoprobe (Varian, Palo Alto, CA, USA). Chemical shifts are referenced to deuterated water (D_2_O) peaks and reported in ppm [27].

DSC analysis was performed using differential scanning calorimetry (DSC 214 Polyma, Netzsch, Germany). For this analysis, 10 mg of respective samples were weighed in new aluminium crucibles and placed in the DSC furnace with an empty aluminium crucible as the reference. The samples were purged with nitrogen gas and heated at a constant heating rate of 10 °C/min over a temperature range of − 20 to 200 °C [9, 15]. Two heating cycles with intermediate cooling were performed to avoid thermal history, and only the spectrum obtained during the second heating cycle is reported in the present study.

#### Liquid and gas phase analysis of dark fermentation

Before collecting the gas samples, the pressure in the headspace was measured using a handheld pressure gauge (Keller Druckmesstechnik Mano Leo1 81000.2, Jestetten, Germany). The volume of biogas produced in the headspace was calculated by correlating the pressure value with the ideal gas law [19]. Biogas composition was analysed by using a gas chromatograph fitted with a thermal conductivity detector (GC, 7890B, Agilent Technologies, Santa Clara, USA) and a Porapak Q (2.74 m × 2 mm) column [19]. Likewise, 2 mL of liquid samples were collected by inverting the bottle, and the samples were filtered using 0.22 μm cellulose acetate filters before liquid chromatography and total carbon (TC) analysis. Filtered samples were injected into a liquid chromatograph (1260 Infinity II, Agilent, Santa Clara, USA) comprising a Hi-Plex H column and refractive index (RI) detector [19]. The total carbon content of the liquid fraction of the digestate obtained during the dark fermentation was measured using a total organic carbon analyser (TOC-L CSN Analyser, Shimadzu, Japan) as described by Manikandan and Lens [9].

### Microbial community analysis

Microbial samples were withdrawn at the end (36 h incubation) of the experiments, and the DNA of each sample was extracted using a QIAGEN DNeasy power soil kit (QIAGEN, Hilden, Germany) as per the protocol disclosed by the manufacturer. The concentration of the extracted DNA was measured using a Qubit fluorometer (Invitrogen, Carlsbad, CA, USA), and the samples were stored at − 20 °C until further use [28]. Amplifying DNA samples using polymerase chain reaction (PCR) was carried out using specific universal primers (515F–806R) that amplify the V4 region of samples. This amplification and further purification, library preparation, sequencing and bioinformatic analysis were carried out at the Novogene Institute (Beijing, China) as described in detail by Jiang et al. [29].

## Results and discussion

### Biorefining of ulvan from *Ulva* spp. using citric acid as the extractant

Using citric acid extraction, a total ulvan yield of 0.27 g of ulvan from one gram of dry *Ulva* spp. biomass was obtained, while generating 0.68 g of ulvan-free biomass as the refuse from the ulvan extraction process. Previously, at the same extraction conditions of 3 wt% of *Ulva* spp. biomass, 1wt% of citric acid as extractant and at an extracting temperature of 90 °C resulted in a more elevated ulvan yield of 0.41 (± 0.02) g/g [9]. The variation in the ulvan yield of 0.41 g/g versus 0.27 g/g in the present study can be attributed to differences in the chemical composition, which is strongly dependent on the harvesting period of the *Ulva* spp. biomass. The seasonal variability of the ulvan content in *Ulva* spp. biomass at the summer's beginning, mid and end is well-documented in the literature [30]. The present study addresses the seasonal variation of *Ulva* spp. in proposing to use ulvan-free biomass to produce more biopolymer, viz. polyhydroxybutyrate, under a zero-waste discharge approach.

**Fig. 1:**
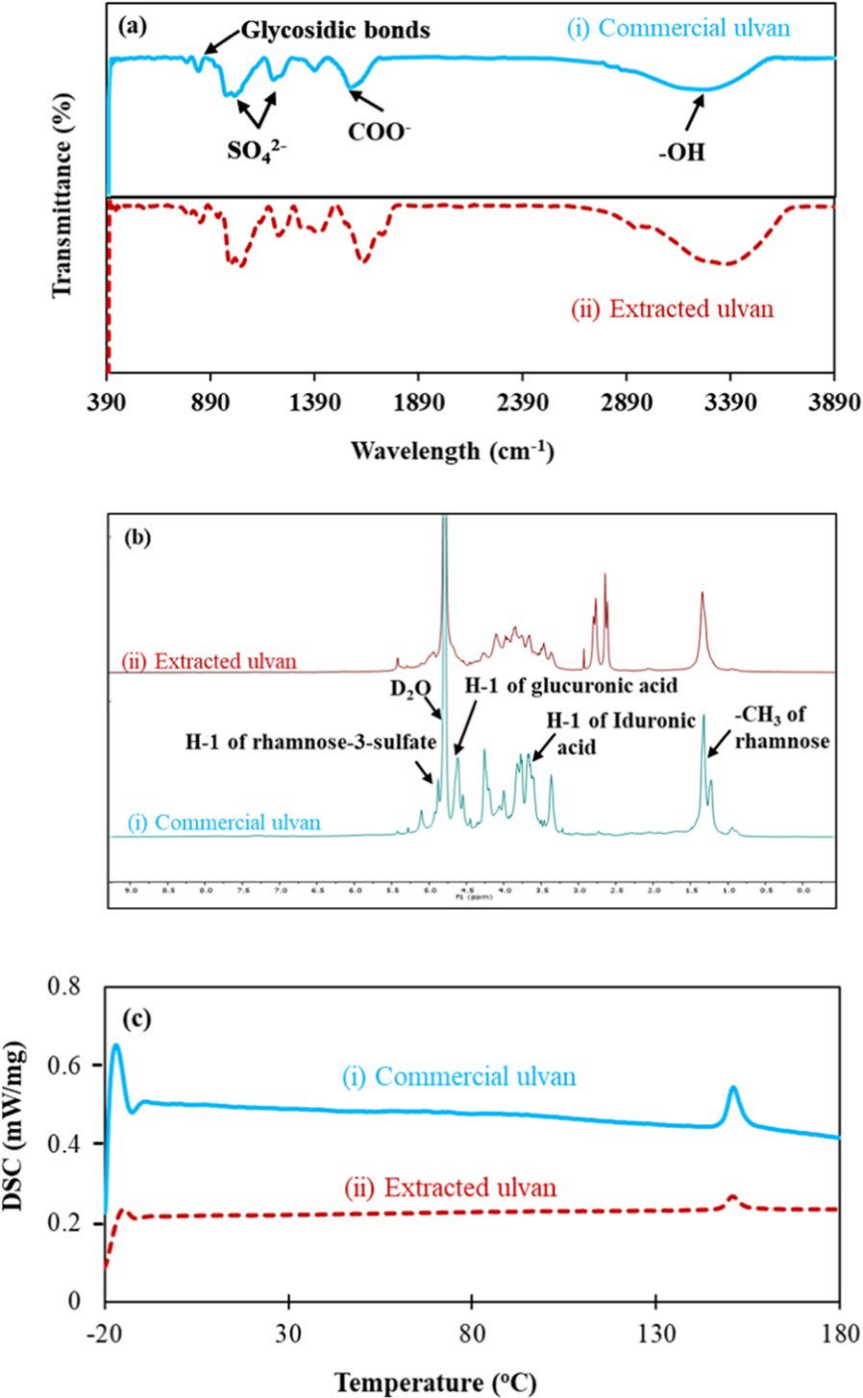
Comparison of ulvan extracted (i) in the present study to that of the commercial ulvan (ii) using: **a** FTIR, **b** Proton-NMR and **c** DSC analysis

FTIR analysis revealed the similarity of the extracted ulvan to that of the commercial ulvan by matching the fingerprint region of sulfated polysaccharides observed due to the stretching vibration of sulfate around 1028 and 1211 cm^−1^. Likewise, carboxyl (COO^−^) and hydroxyl (-OH) groups were also matched with a band at 1628 and 3362 cm^−1^, respectively (Fig. 1a). The predominant molecules found in ulvan, viz. rhamnose and uronic acids, were revealed in the proton-NMR analysis with a chemical shift observed at 4.8 and 3.8 ppm, respectively (Fig. 1b). This chemical shift matches well with the chemical shifts observed in the commercial ulvan and the results reported in the literature by Yaich et al. [27]. The additional shifts observed around 2.5 ppm may stem from a slight protein intrusion into the ulvan extract. The intrusion of a negligible amount of protein into the ulvan extract was previously reported by Manikandan and Lens (2022) [9]. The DSC analysis portrays the melting temperature of both the commercial and extracted ulvan matched well at 151 °C (Fig. 1c).

### Edible ulvan films and their effect on gut-friendly and pathogenic microbes

Ulvan films have previously been formed by plasticisation with sorbitol and glycerol [10]. However, xylitol is a well-known plasticiser and sweetener [31, 32], and its blending with ulvan has not yet been reported in the literature. Therefore, the dual added advantages of xylitol were harnessed in the present study by blending ulvan with xylitol. Further, citric acid, a widely used crosslinking agent to strengthen water-soluble polymers, was added as a tertiary compound to prepare the ulvan films [9, 15].

**Fig. 2:**
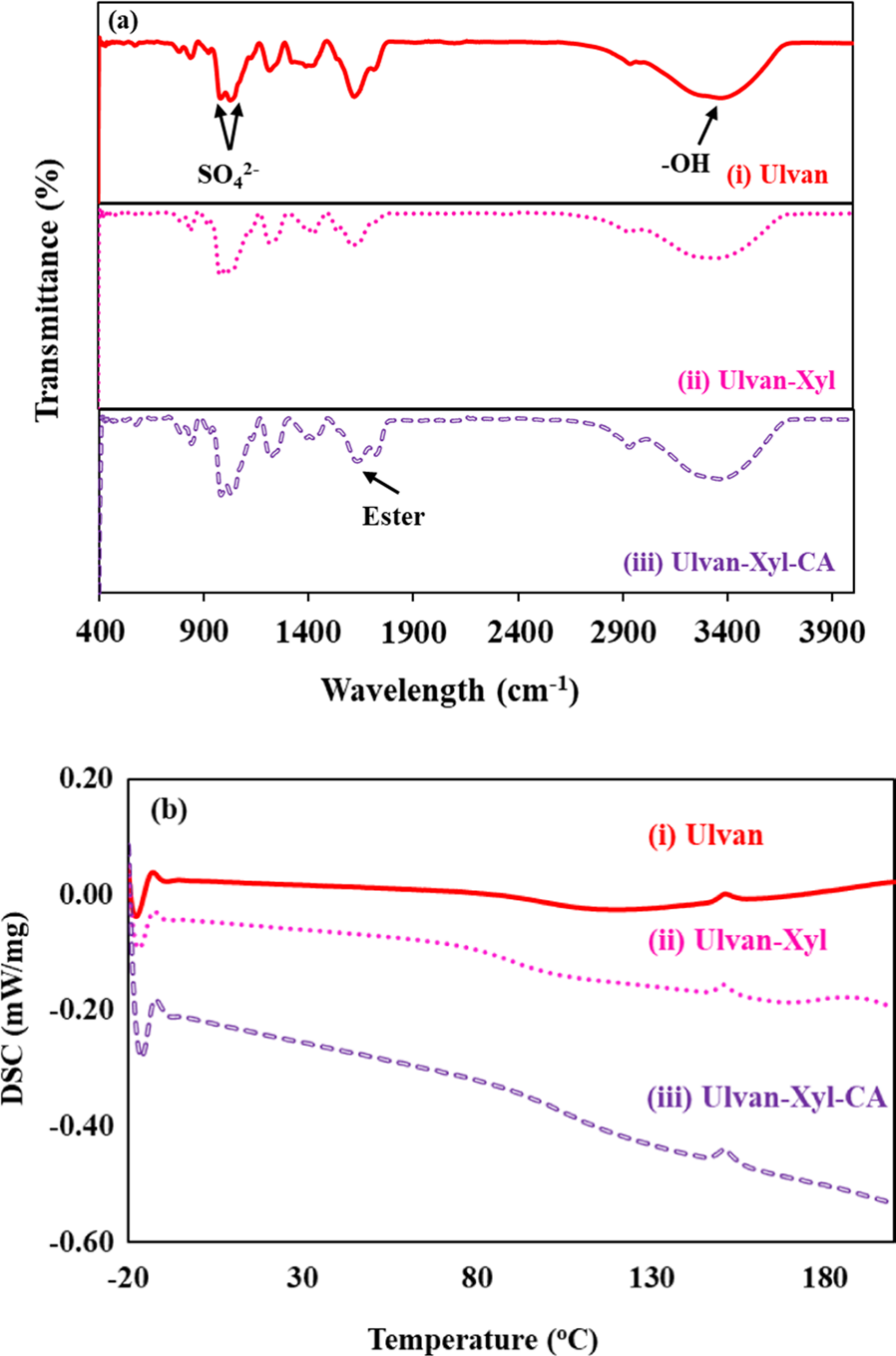
FTIR (**a**) and DSC (**b**) analysis of various edible films, namely (i) Ulvan, (ii) Ulvan-Xyl and (iii) Ulvan-Xyl-CA

FTIR analysis revealed that the blending of xylitol or citric acid did not change the sulfated backbone structure of the ulvan observed around 1027 cm^−1^ (Fig. 2a). Similarly, the inclusion of xylitol into the ulvan showed no changes in the FTIR spectrum (Fig. 2a). The polyester formation in ulvan noticed upon the addition of citric acid was revealed with the origination of a new ester bond at 1720 cm^−1^ (Fig. 2a). This ester formation due to crosslinking of water-soluble polymers like carboxymethyl cellulose (CMC), hydroxypropyl methylcellulose (HPMC) and starch has been previously reported in the literature [15]. Retaining of the ulvan structure was confirmed in the DSC analysis, wherein the melting point of all three edible films, viz. ulvan, ulvan-Xyl and Ulvan-Xyl-CA are 150.96, 150.96 and 151.46 °C, respectively (Fig. 2b). It's noteworthy to retain such an elevated melting point, as edible packaging films should have such an inherent property to handle warm foods.

**Fig. 3:**
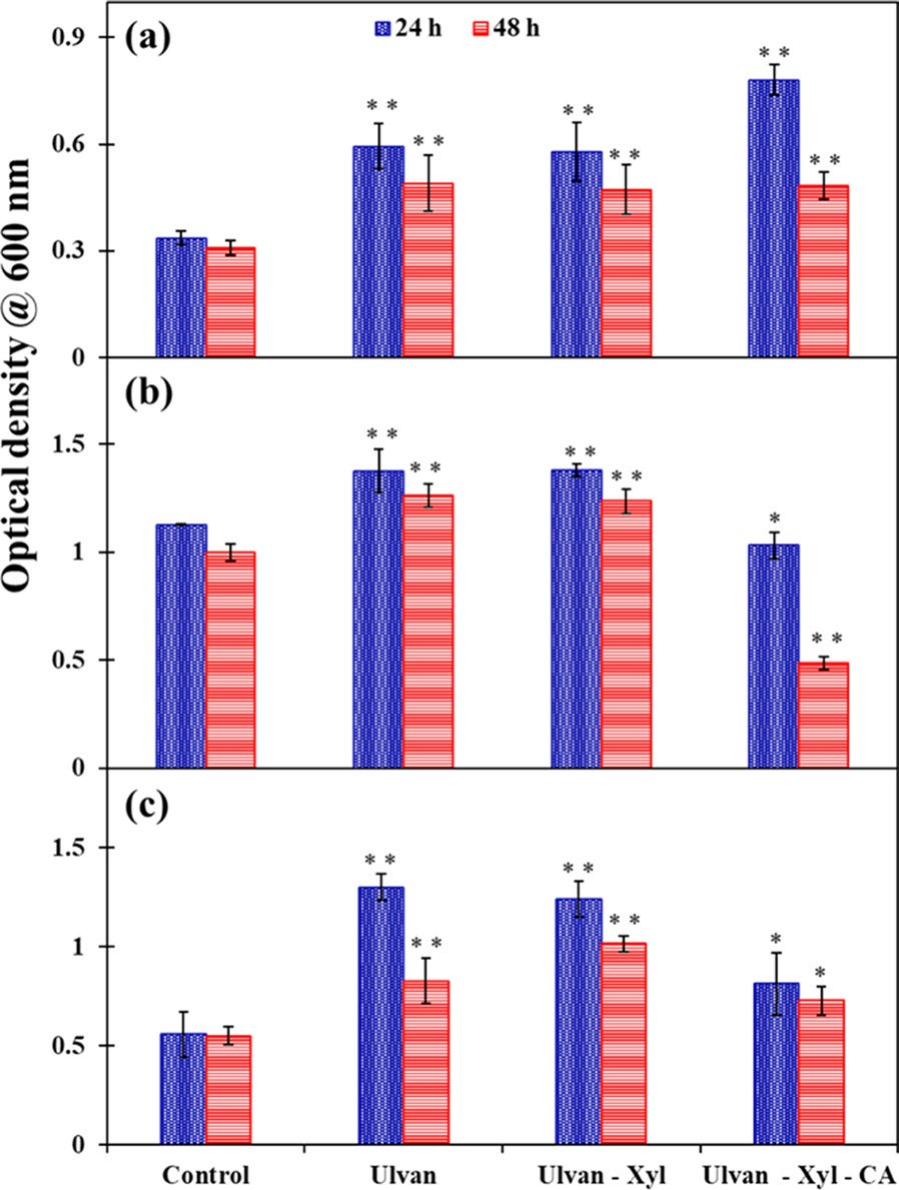
Microbial growth in the presence of various edible films, viz. ulvan, ulvan-xyl and ulvan-xyl-CA, carried out using: **a** gut-friendly microbiota, **b** *E. coli* and **c** *S. aureus*. Using Tukey’s test, the statistically significant differences at P < 0.05 and statistically insignificant variation was marked with ** and *, respectively

In addition to taste and texture, the edible nature of a packaging film is highlighted by its performance in boosting the gut microbiome [33]. Figure 3 portrays that irrespective of the films tested in the present study, the optical density of gut-friendly microbiota (yoghurt) as well as pathogens (*E. coli* and *S. aureus*) are enhanced compared to the control experiment containing no films added to the test tubes. For instance, the OD of 0.34 (± 0.02) observed in the control experiment for gut-friendly microbiota increased to 0.59 (± 0.06), 0.58 (± 0.08) and 0.78 (± 0.04) in the presence of ulvan, ulvan-xyl and ulvan-xyl-CA films, respectively (Fig. 3a). Likewise, the OD of 1.13 (± 0.03) observed in the control experiment for *E. coli* was increased to 1.38 (± 0.1) and 1.38 (± 0.03) in the presence of ulvan and ulvan-xyl films, respectively (Fig. 3b). In the case of *S. aureus*, a similar trend was observed, wherein the OD of 0.56 (± 0.11) observed in the control experiment increased to 1.30 (± 0.07) and 1.24 (± 0.09) in the presence of ulvan and ulvan-xyl films, respectively (Fig. 3c). Unlike the gut-friendly microbiota, the presence of citric acid in the edible films retarded the growth of *E. coli* and *S. aureus* with an OD of 1.03 (± 0.06) and 0.81 (± 0.16), respectively. The stronger growth inhibition of *E. coli* compared to that of *S. aureus* in the presence of ulvan-xyl-CA film can be attributed to the absence or presence of a peptidoglycan layer in gram-negative (*E. coli*) versus gram-positive bacteria (*S. aureus*), respectively [21].

**Fig. 4:**
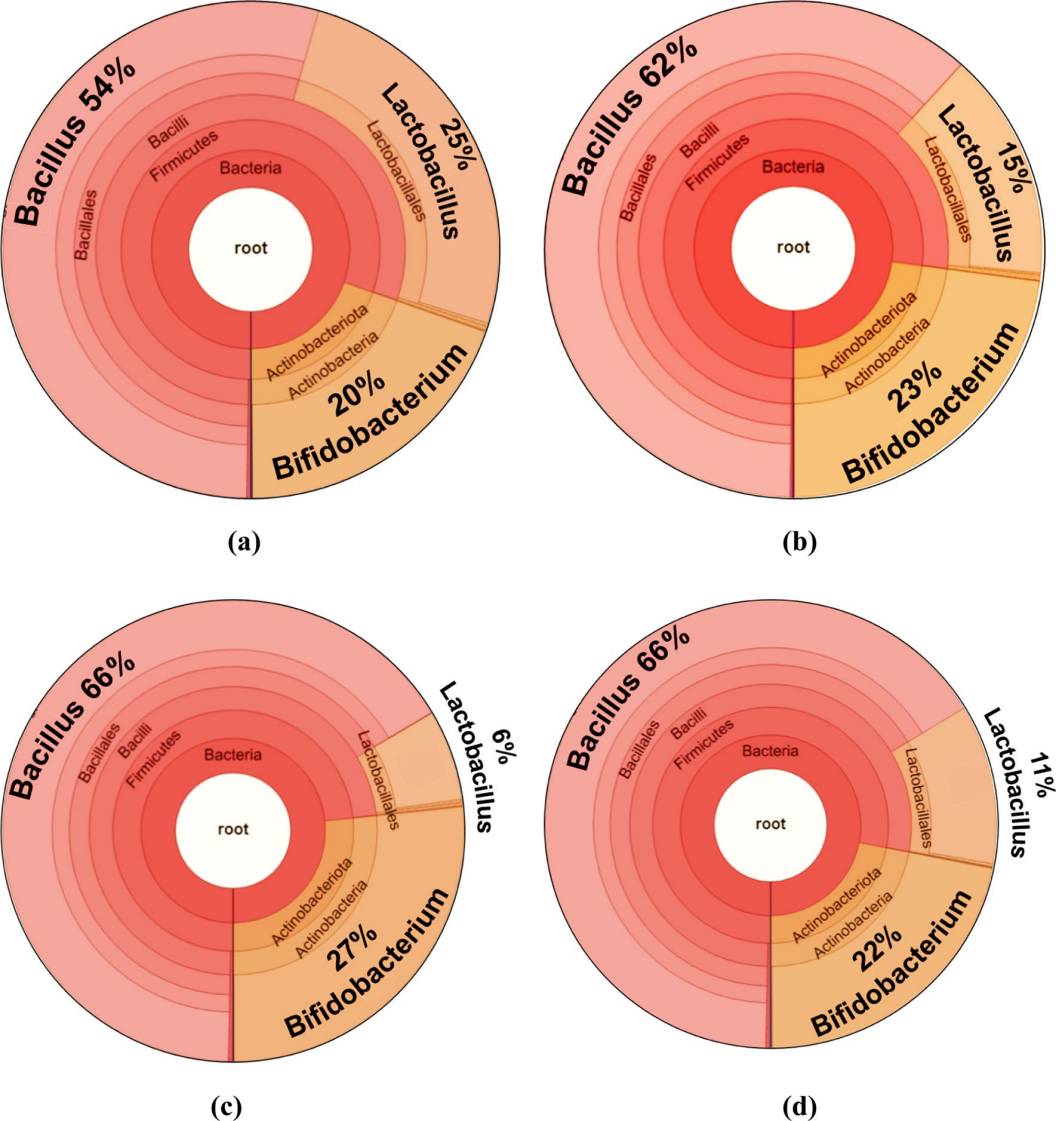
Krona chart displaying the microbial community amongst the gut-friendly microbiota observed at the end of the control experiment (**a**) and experiments carried out in the presence of edible ulvan films, viz. ulvan (**b**), ulvan-xyl (**c**) and ulvan-xyl-CA (**d**)

While ulvan boosted the growth of all microbes, xylitol did not interfere with the growth of microbes (Fig. 3). Interestingly, citric acid increased the growth of the gut-friendly bacteria, while playing an antimicrobial role in retarding the growth of the pathogens. This antimicrobial activity of citric acid against *E. coli* and *S. aureus* was previously reported by Dharmalingam and Anandalakshmi (2019) and Koneru et al. [15, 34]. Further, the microbial community analysis showed that xylitol didn't enhance the growth of microbes. In contrast, the presence of xylitol in an ulvan-xyl film selectively enhanced the population of *Bifidobacterium* sp. to a relative abundance (RA) of 27% compared to the 20% RA of *Bifidobacterium* sp. observed in the control (Fig. 4 a and c). Likewise, reducing the xylitol concentration and replacing it with citric acid in an ulvan-xyl-CA film reduced the RA of the *Bifidobacterium* sp. from 27 to 22% (Fig. 4c). The enhancement in a *Bifidobacterium* sp. population consequently resulted in the reduction of the RA of *Lactobacillus* sp. (Fig. 4 c and d). This result of enhancement of *Bifidobacterium* sp. in the presence of xylitol agrees with the literature report by Xiang et al. (2021) [35], who deduced the mechanism of the action of xylitol in promoting the proliferation of beneficial bacteria in the colon.

### Dark fermentation of ulvan-free *Ulva* spp. for VFAs production

**Fig. 5:**
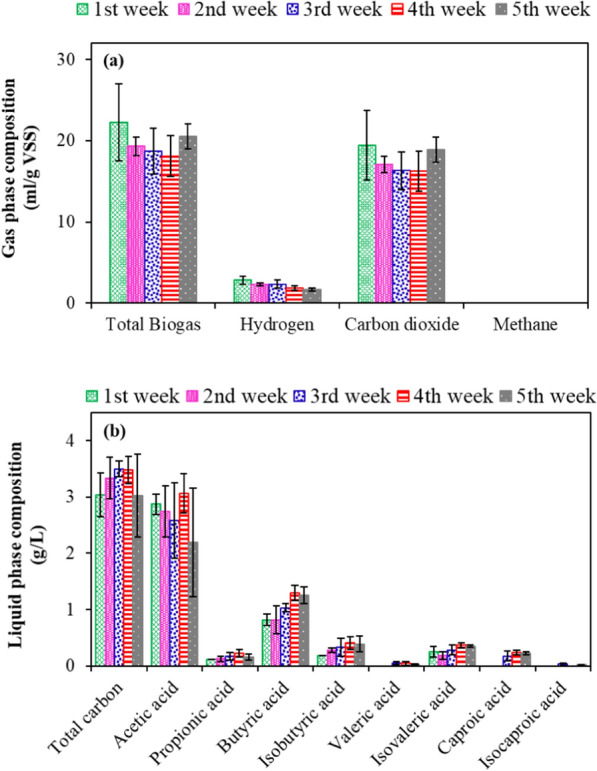
Gas phase (**a**) and liquid phase (**b**) composition monitored during the dark fermentation (pH of 7 and temperature of 37 °C) of ulvan-free *Ulva* biomass following the substrate overloading strategy (Inoculum/substrate ratio of 1:5)

Dark fermentation at a high substrate loading rate (inoculum/substrate ratio of 1:5) and an extended incubation time of five weeks was carried out to divert towards maximum VFAs production. As expected, the extended incubation period led to the utilisation of hydrogen for VFAs production, leaving scarce hydrogen of 2.86 (± 0.49) ml/g VSS in the headspace in the first week (Fig. 5). This value of hydrogen steadily declined to 1.67 (± 0.18) ml/g VSS in the fifth week. The inoculum pretreatment resulted in an unnoticeable amount of methane in the headspace (Fig. 5a). Further, the krona chart shown in Fig. 5a portrays the microbial consortia predominantly consisted of hydrogenogens like *Clostridiales* (74%) and *Peptostreptococcales* (19%) instead of methanogens.

**Fig. 6:**
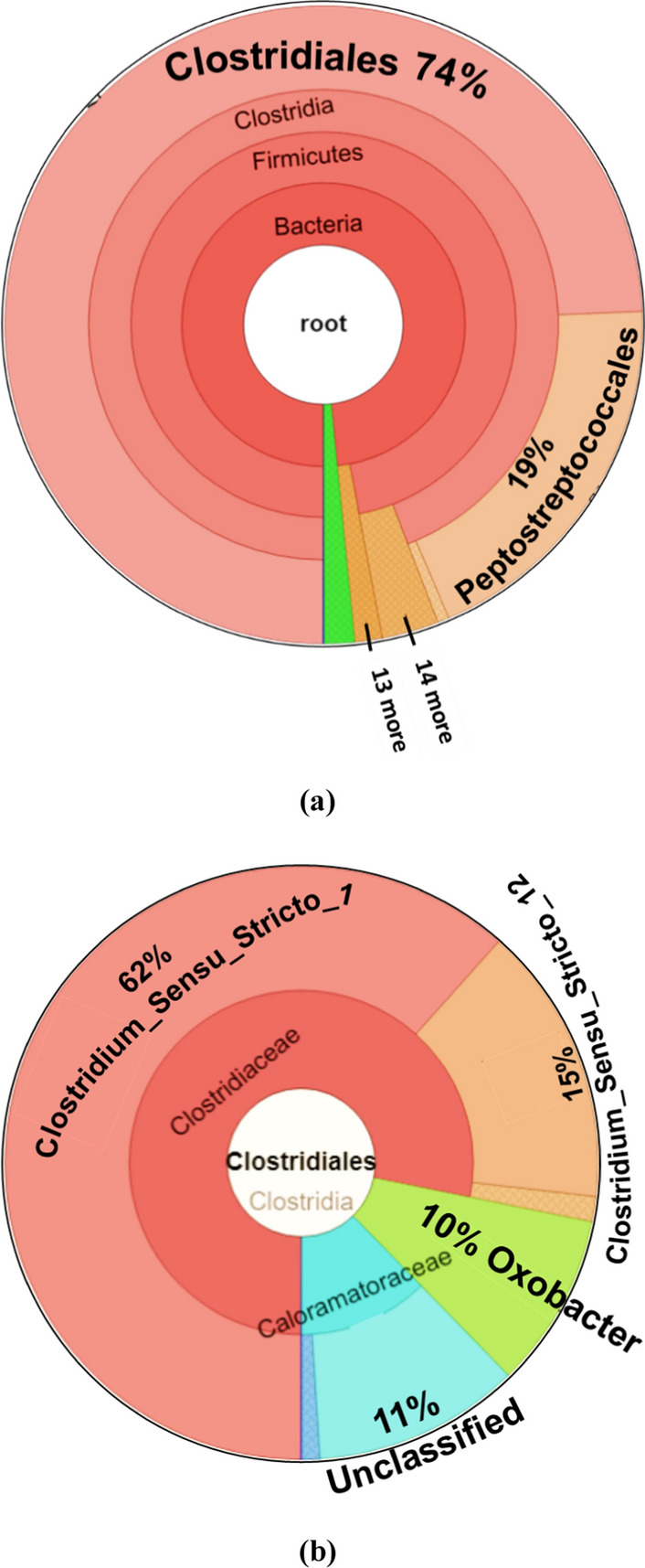
Krona chart displaying the microbial community observed at the end of the dark fermentation experiments (5th week of incubation): **a** species level analysis and **b** strain level analysis carried out with a specific focus on the clostridiales

These aforementioned species are well known for hydrogen production and carbon dioxide conversion to volatile fatty acids. More specifically, *Peptostreptococcales* (Fig. 6a) are known VFA producers and *Oxobacter* (Fig. 6b) is known for chain elongation from acetic acid to butyric and iso-butyric acid [36, 37]. These microbes are thus expected to have played a key role in producing VFAs, containing a total carbon of 3.03 (± 0.12) g/L at the end of the first week (Fig. 5b). Acetic and butyric acid was predominantly observed at a concentration of 2.87 (± 0.11) g/L and 0.82 (± 0.03) g/L at the end of the first week, respectively (Fig. 5b). Increasing the incubation period of dark fermentation from 1 to 5 weeks did not significantly increase the VFAs concentration. However, the shorter chain VFAs were converted to medium chain fatty acids like isovaleric and caproic acid with a maximum concentration of, respectively, 0.35 (± 0.014) g/L and 0.22 (± 0.01) g/L at the end of the fifth week. This pattern of caproic acid production at long-term operation and acetic acid production at short-term operation was previously reported by Owusu-Agyeman et al. (2020) [38] during co-digestion of sewage sludge with external organic waste.

### Polyhydroxybutyrate production using *C. necator* fed with VFAs-rich digestate

**Fig. 7:**
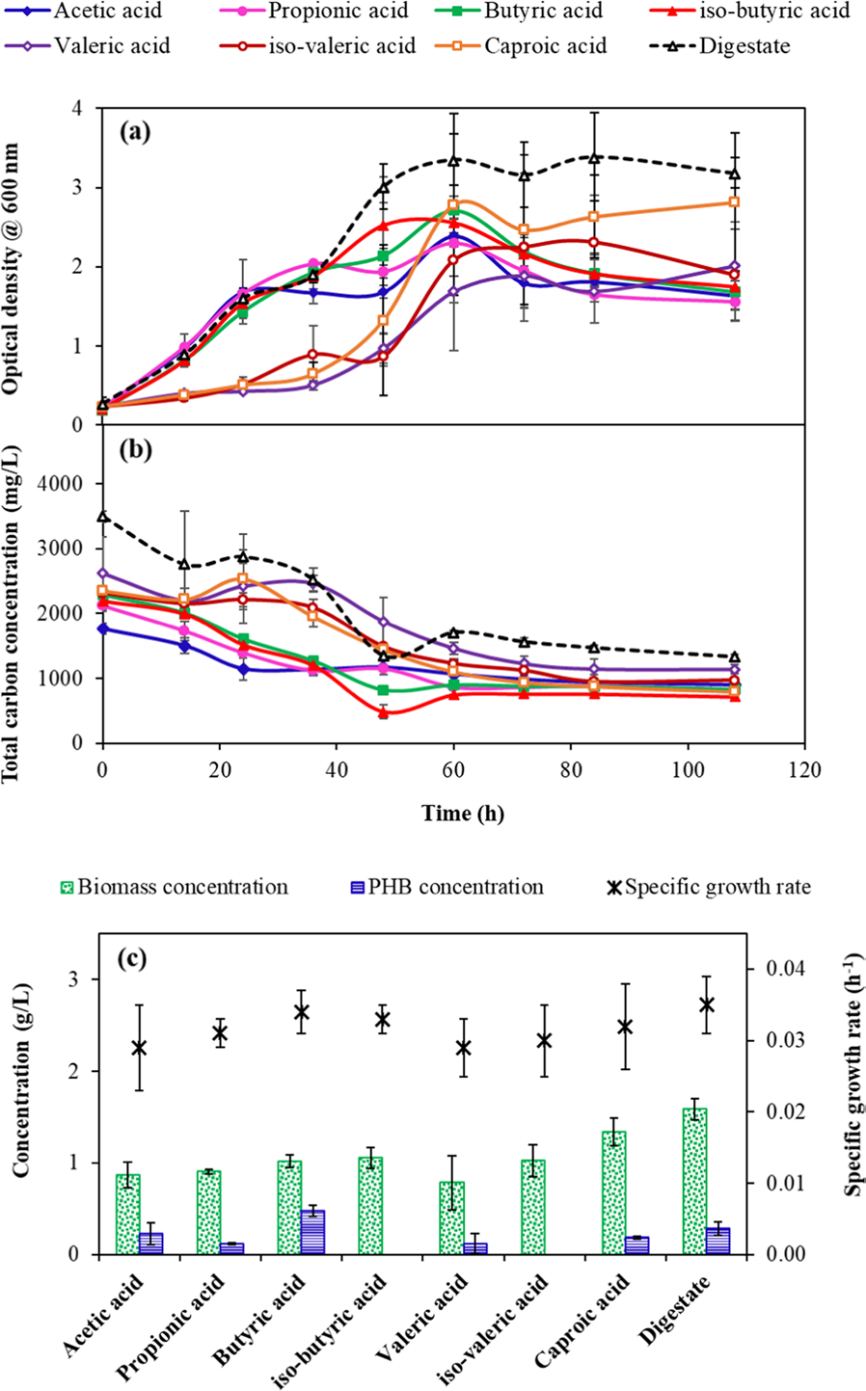
Biomass growth (**a**) and substrate consumption (**b**) of *C. necator* using different synthetic VFAs as the carbon source and the VFAs-rich liquid fraction of the digestate obtained at the end of the dark fermentation of ulvan-free biomass. *C. necator* biomass and PHB concentration observed at the end of the aerobic experiments and its specific growth rate (µ) are shown in **c**

Figure 7 shows the biomass growth (Fig. 7a) and substrate utilisation (Fig. 7b) kinetics of *C. necator* grown using different VFAs and the liquid fraction of the digestate originating from the dark fermentation. The liquid fraction of the digestate contained acetic acid 2.2 (± 0.09) g/L, propionic acid 0.15 (± 0.01) g/L, butyric acid 1.25 (± 0.05) g/L, iso-butyric acid 0.39 (± 0.02) g/L, valeric acid 0.02 (± 0.001) g/L, iso-valeric acid 0.35 (± 0.01) g/L, caproic acid 0.22 (± 0.01) g/L and iso-caproic acid 0.016 (± 0.001) g/L. The *C. necator* biomass concentration when grown on medium-chain fatty acids like caproic acid (1.34 ± 0.15 g/L), was higher than the *C. necator* biomass concentration grown with short-chain fatty acids like acetic acid (0.87 ± 0.14 g/L) and propionic acid (0.91 ± 0.02 g/L) (Fig. 7c).

However, the polyhydroxybutyrate content of the *C. necator* biomass was high only when *C. necator* was grown on butyric acid (Fig. 7c). For instance, *C. necator* fed with butyric acid as the sole carbon source resulted in a maximum PHB concentration of 0.48 (± 0.06) g/L and a PHB yield of 0.14 (± 0.02) g/g of VFA (Fig. 7c and Table 1). Likewise, the inherent presence of an elevated butyric acid (1.25 ± 0.05 g/L) in the liquid fraction of the digestate resulted in a PHB concentration of 0.29 (± 0.001) g/L (Fig. 7c). Further, a high specific growth rate of 0.034 (± 0.001) h^−1^ was observed in the case of *C. necator* fed with butyric acid as the sole carbon source. Thus, strategies to transform acetic acid to butyric acid would be very beneficial for polyhydroxybutyrate production using *C. necator*. In the present study, a longer incubation time of the dark fermentation process yielded a higher butyric acid concentration.

**Table 1:** Biomass and PHB yield of *C. necator* grown using different synthetic VFAs as the carbon source and the VFAs-rich liquid fraction of the digestate obtained at the end of the dark fermentation of ulvan-free biomass

Substrate	Biomass yield (g of biomass/ g of VFA)	PHB yield (g of PHB/ g of VFA)	Specific growth rate (h^−1^)
Acetic acid	0.31 ± 0.07	0.07 ± 0.03	0.029 ± 0.01
Propionic acid	0.37 ± 0.06	0.03 ± 0.001	0.031 ± 0.001
Butyric acid	0.4 ± 0.03	0.14 ± 0.02	0.034 ± 0.001
Iso-butyric acid	0.36 ± 0.07	–	0.03 ± 0.001
Valeric acid	0.22 ± 0.08	0.04 ± 0.03	0.029 0.004
Iso-valeric acid	0.29 ± 0.05	–	0.03 ± 0.005
Caproic acid	0.38 ± 0.04	0.05 ± 0.001	0.032 ± 0.006
Digestate	0.43 ± 0.02	0.06 ± 0.02	0.04 ± 0.001

**Fig. 8:**
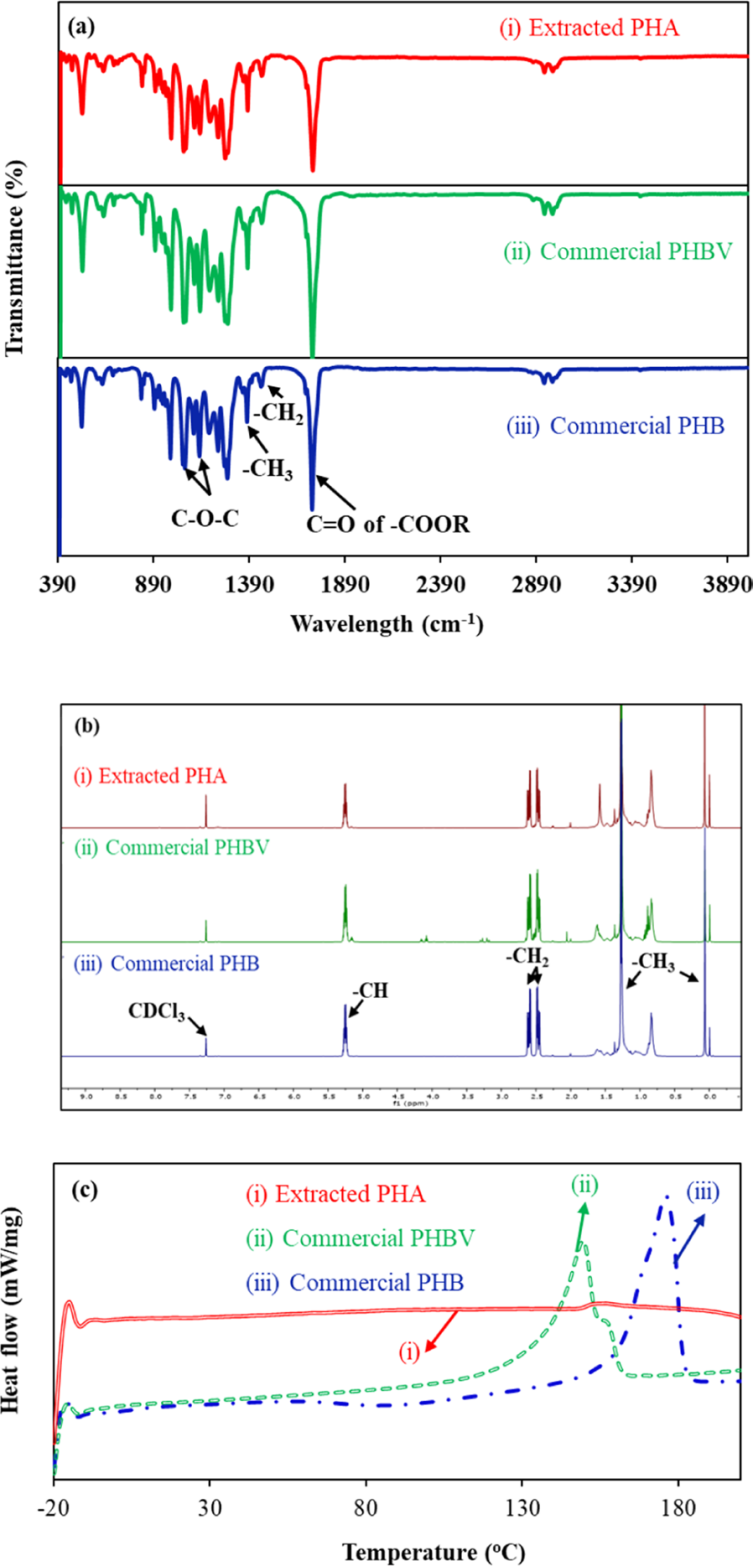
Comparison of polyhydroxyalkanoate (PHA) extracted from *C. necator* grown using the liquid fraction of digestate produced during the dark fermentation of ulvan-free biomass in the present study (i) to that of commercially available PHBV (ii) and PHB (iii) using: **a** FTIR, **b** Proton-NMR and **c** DSC analysis

The PHA extracted from *C. necator* cells grown using VFAs from the liquid fraction of the digestate from dark fermentation was compared with the commercially available PHB and PHBV using FTIR, Proton-NMR and DSC analysis (Fig. 8). Polyhydroxyalkanoate being a polyester, the carbonyl (C=O) group in the ester (-COOR) was prominently seen at 1727 cm^−1^ in both the extracted PHA and commercially available PHB/PHBV. Likewise, C-H bonds stemming from –CH_2_ and –CH_3_ in polyhydroxyalkanoate were noticed on the extracted PHA and commercial PHB/PHBV at 1460 and 1380 cm^−1^, respectively (Fig. 8a). The C–O–C stretching was observed between 100 to 1300 cm^−1^, which agrees with the results of Dhangdhariya et al. (2015) on polyhydroxyalkanoates produced using dry sea mix. Proton-NMR analysis matched the three signals due to methyl (‒CH_3_), methylene (‒CH_2_) and methine (‒CH) groups (Fig. 8b). For instance, the signal with its chemical shift at 5.2 ppm denotes the methine group, two multiplet signals were observed at 2.55 ppm, and doublets corresponding to the methylene group were noticed in the extracted and the commercial PHA (Fig. 8b). Similar chemical shifts have been widely reported for the proton-NMR analysis of polyhydroxyalkanoates produced by various microbes, including *Bacillus subtilis*, *Bacillus megaterium* and *Vibrio harveyi* [39, 40]. The DSC analysis depicted that the melting point (154 °C) of extracted PHA matched closely with the melting point (150 °C) of the PHBV copolymer rather than that of the PHB, which exhibited its melting point at 177 °C (Fig. 8c). Further, the well-defined peak in the commercial PHB portraying the well-organised polymeric chains and the crystalline nature of the biopolymer was absent in the case of extracted PHA. The shallow peak noticed in the case of extracted PHA can be attributed to the amorphous nature of the polymer. However, further study must be performed using X-ray diffraction (XRD) analysis to conclude the crystallinity of the extracted PHA.

### Practical applicability and future directions to develop a seaweed biorefinery

**Fig. 9:**
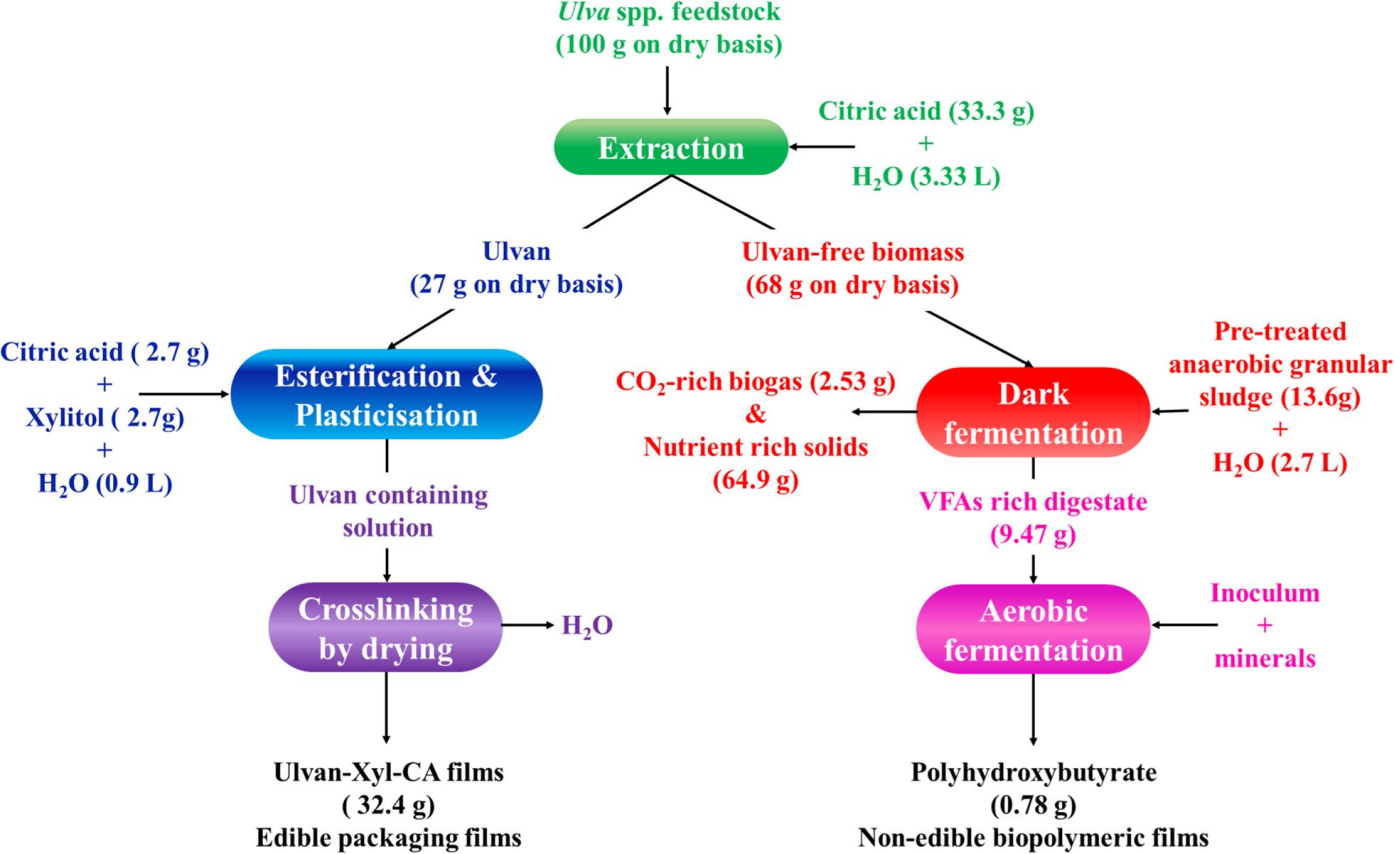
Mass balance for producing edible ulvan and non-edible polyhydroxybutyrate biopolymeric films using *Ulva* spp. as the sustainable feedstock

Figure 9 shows the mass balance analysis for the sustainable biorefining and bioprocessing strategy handled for producing edible-ulvan and non-edible polyhydroxybutyrate biopolymeric films using 100 g of *Ulva* spp. as the feedstock. It can be noticed that the yield of edible ulvan films (32.4 g) was higher than the yield of polyhydroxybutyrate (1.18 g) films. The very high yield of the edible-ulvan film is attractive as it can act as a future food supplement and a sustainable packaging solution resulting in no packaging waste.

The rather low PHB yield can be attributed to the fact that 68 g of ulvan-free biomass was converted to only 9.47 g of VFAs and thereafter to only 4.33 g of dry *C. necator* cells containing 18.2 wt% of PHA. Further optimisation of the dark fermentation step by adding suitable minerals, e.g., iron and nickel [41], nutrients or static magnetic fields [42] to boost the pre-treated AGS biomass for VFAs production is required. Further, the scanty PHB content in *C. necator* cells could be due to the elevated amount of nitrogen source stemming from the protein present in the ulvan-free biomass. The nitrogen source plays a vital role in PHB accumulation, as *C. necator* cells accumulate PHB only at nitrogen-deprived culture conditions [43]. Thus, the co-feeding strategy of adding a higher amount of carbon source to the digestate, as indicated by Li et al. [44] needs to be investigated in the future.

In addition, 1.4 L of carbon dioxide-rich biogas was produced during dark fermentation, which was calculated to be 2.52 g of carbon dioxide and 0.01 g of hydrogen at STP conditions (Fig. 9). In a circular bioeconomy context, this substantial amount of carbon dioxide should be considered for producing ethanol [45] or succinic acid [46] or as a carbon source to promote algal growth [47]. Algal biosolids are well-known to play the role of biofertilisers by enhancing the crop health or plant growth [48]. Thus, the sustainable biorefining and bioprocessing proposed for producing edible and non-edible films can be further developed by optimising the bioprocesses and further investigated for producing other value-added materials, like carbonated water or biofertilizers.

## Conclusions

A novel process was developed for the complete valorisation of *Ulva* spp. by producing edible ulvan films and non-edible polyhydroxyalkanoates. Edible ulvan films prepared by crosslinking with citric acid and plasticisation using xylitol promoted the growth of gut-friendly microbiota, while retarding the growth of pathogens. The macroalgal biomass refused after ulvan extraction was strategically converted to volatile fatty acids by dark fermentation. The liquid fraction of the digestate enriched with VFAs was used to produce non-edible polyhydroxyalkanoates using *C. necato*r. Thus, a zero-waste discharge approach was developed by biorefining *Ulva* spp. to extract ulvan and bioprocessing ulvan-free biomass for polyhydroxyalkanoates production.

## Data Availability

All data were provided in the manuscript.

## Abbreviations

FTIR: Fourier transform infrared spectroscopy
DSC: Differential scanning calorimetry
NMR: Nuclear magnetic resonance
VFAs: Volatile fatty acids
Xyl: Xylitol
CA: Citric acid
GC: Gas chromatography
HPLC: High-pressure liquid chromatography
PHB: Polyhydroxybutyrate
PHBV: Polyhydroxybutyrate valerate
AGS: Anaerobic granular sludge
EDTA: Ethylenediaminetetraacetic acid
OD: Optical density
CdCl_3_: Deuterated chloroform
ATR: Attenuated total reflectance
D_2_O: Deuterated water
